# Bioengineered 3D nanocomposite based on gold nanoparticles and gelatin nanofibers for bone regeneration: in vitro and in vivo study

**DOI:** 10.1038/s41598-021-93367-6

**Published:** 2021-07-06

**Authors:** Hadi Samadian, Hossein Khastar, Arian Ehterami, Majid Salehi

**Affiliations:** 1 Nano Drug Delivery Research Center, Health Technology Institute, Kermanshah University of Medical Sciences, Kermanshah, Iran; 2 Sexual Health and Fertility Research Center, Shahroud University of Medical Sciences, Shahroud, Iran; 3 Institute for Regenerative Medicine, University of Zurich, Zurich, Switzerland; 4 Department of Tissue Engineering, School of Medicine, Shahroud University of Medical Sciences, Shahroud, Iran; 5 Tissue Engineering and Stem Cells Research Center, Shahroud University of Medical Sciences, Shahroud, Iran

**Keywords:** Nanoscience and technology, Nanomedicine

## Abstract

The main aim of the present study was to fabricate 3D scaffold based on poly (l-lactic acid) (PLLA)/Polycaprolactone (PCL) matrix polymer containing gelatin nanofibers (GNFs) and gold nanoparticles (AuNPs) as the scaffold for bone tissue engineering application. AuNPs were synthesized via the Turkevich method as the osteogenic factor. GNFs were fabricated by the electrospinning methods and implemented into the scaffold as the extracellular matrix mimicry structure. The prepared AuNPs and Gel nanofibers were composited by PLLA/PCL matrix polymer and converted to a 3D scaffold using thermal-induced phase separation. SEM imaging illustrated the scaffold's porous structure with a porosity range of 80–90% and a pore size range of 80 to 130 µm. The in vitro studies showed that the highest concentration of AuNPs (160 ppm) induced toxicity and 80 ppm AuNPs exhibited the highest cell proliferation. The in vivo studies showed that PCL/PLLA/Gel/80ppmAuNPs induced the highest neo-bone formation, osteocyte in lacuna woven bone formation, and angiogenesis in the defect site. In conclusion, this study showed that the prepared scaffold exhibited suitable properties for bone tissue engineering in terms of porosity, pore size, mechanical properties, biocompatibility, and osteoconduction activities.

## Introduction

Bone fractures are threatening condition which every individual may encounter in his/her life. Although simple fractures heal spontaneously with routine treatment, severe fractures required complicated therapies. The gold standard treatment for severe and extensive fractures is an autograft, which has several drawbacks despite its advantages. Hence, seeking proper alternatives is the subject of various studies and researchers. In this regard, 3D structured nanocomposites have gained a great deal of attention as the bone tissue engineering scaffold due to their remarkable properties and performance^1–3^. Well-designed and fabricated 3D structures can fill bone fractures, prevent collapsing surrounding tissues, and support bone cell proliferation and infiltration^4^. Various techniques have been developed for 3D scaffolds fabrication, such as wet electrospinning, gas-foaming, particulate leaching, rapid prototype-based techniques, and thermally-induced phase separation (TIPS) method. TIPS can be highly applicable among these methods due to its relative simplicity, flexibility, and high output^5,6^.

TIPS technique provides 3D structured scaffold with adjustable pore size and pore interconnectivity, which are critical for bone tissue regeneration. Moreover, this method allows the combination of materials with distinct structures to fabricate composite scaffolds. During the TIPS process, the homogenous polymer solution undergoes a phase separation under proper thermal situation resulting in the formation of polymer-rich and polymer-lean phases^7,8^. Using this method, it is possible to fabricate bioactive and functional 3D scaffolds with a combination of bioactive and structural materials. A sophisticated scaffold should mimic the native structure of the host tissue as much as possible. Nanofibrous scaffolds are desirable in this concept, which resembles the extracellular of native tissues^9^. Generally, traditional nanofibers fabrication methods such as electrospinning produce 2D structures that are not favorable for bone tissue engineering. An alternative approach is the combination of these methods to insert nanofibrous features into a 3D scaffold^3^.

Gelatin is a biopolymer obtains from the hydrolysis of collagen, which exhibited fascinating activity favorable for tissue engineering. Gelatin offers attractive properties such as biocompatibility, biodegradability, low cost, low immunogenicity, and acceptable solubility^10^. Moreover, it possesses several RGD (Arg-Gly-Asp) domains in its structure, which is beneficial for cell attachment and proliferation. Electrospun gelatin biomaterials have grabbed significant attention in regeneration medicine. Various studies utilized gelatin nanofibers in pure form or in combination with different biomaterials or structures for bone tissue engineering applications^11,12^. In addition to nanofibers, 3D scaffolds bearing colloidal nanoparticles (NPs) are of interest for tissue engineering applications, which offer a double advantage of having the features of both the NPs and the 3D structures^13,14^.

Gold NPs (AuNPs) have been widely evaluated in various biomedical fields such as cancer therapy, drug/gene delivery, biosensors, cell tracking, and regenerative medicine^15–17^. AuNPs offer brilliant properties such as biocompatibility, chemical inertness and stability, high surface to volume ratio, easy synthesis, and surface modification. It is documented that AuNPs can act as the osteogenic agent and promotes bone cell differentiation and proliferation^18–21^. Yi et al. reported that AuNPs can elicit osteoinductive activity on mesenchymal stem cells (MSCs) via activating the p38 mitogen-activated protein kinase (MAPK) pathway^22^. Bone structure is a natural 3D nanocomposite comprised of collagen nanofibers and hydroxyapatite crystals^23^. An effective scaffold should mimic the bone structure to promote the functions of bone cells. Various types of nanocomposites have been evaluated as scaffolds for bone regeneration, such as nanofibrous nanocomposites, freeze-dried scaffolds composited with NPs or nanofibers, TIPS scaffolds composited with NPs or nanofibers, and hydrogels composited with NPs or nanofibers. To the best of our knowledge, the combination of 3D structures with both NPs and nanofibers in the form of nanocomposite has not been reported in previous studies^24^.

**Scheme 1:**
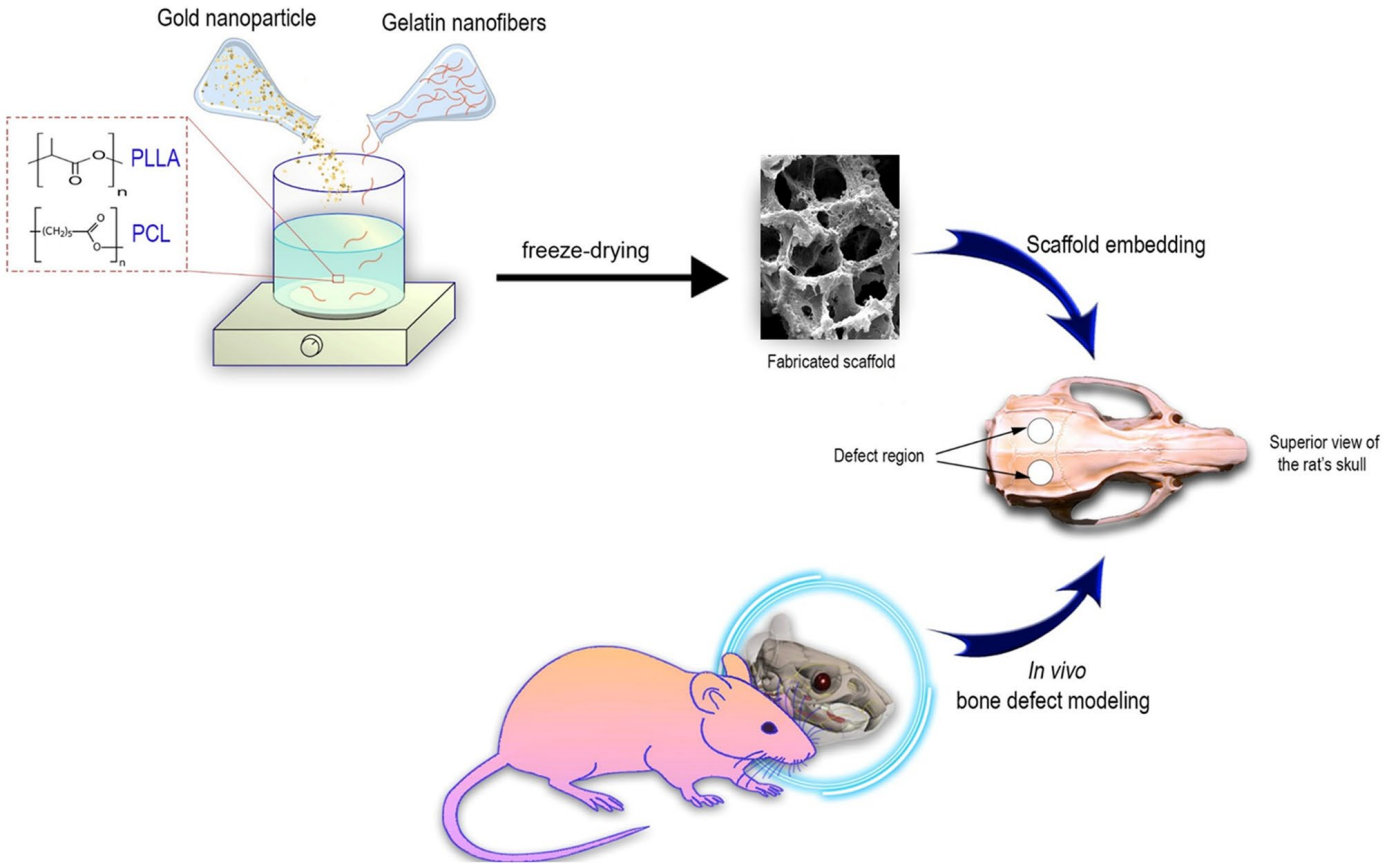
Schematic representation of the study.

Accordingly, in the present study, we fabricated 3D scaffolds based on polylactic acid (PLLA)/Polycaprolactone (PCL) polymers containing Gel nanofibers (GNFs) and AuNPs for bone regeneration. The applied PLLA/PCL served as the matrix, GNFs as the mimicry of the bone extracellular matrix (ECM), and AuNPs as the healing agent (Scheme 1). Different types of scaffolds have been evaluated as bone regenerating materials. 2D scaffolds, such as nanofibers, are promising due to their resemblance to the bone ECM, but they cannot fill the bone defect and properly mimic the 3D structure of the bone cells niche. On the other hand, the conventional 3D scaffolds without resemblance to native bone tissue may not properly improve bone regeneration. Accordingly, the combination of these concepts can beneficiate from the positive features of 2D and 3D scaffolds. The combination of PCL/PLLA/GNFs/AuNPs has not been evaluated in the form of a 3D scaffold for bone regeneration in animal models. The significant novelty of the present study is the combination of GNFs to mimic collagen nanofibers and AuNPs to mimic hydroxyapatite crystals to prepare a sophisticated scaffold with the most resemblance functionality to native bone structure.

## Results and discussion

### Nanocomposite characterization finding

**Figure 1:**
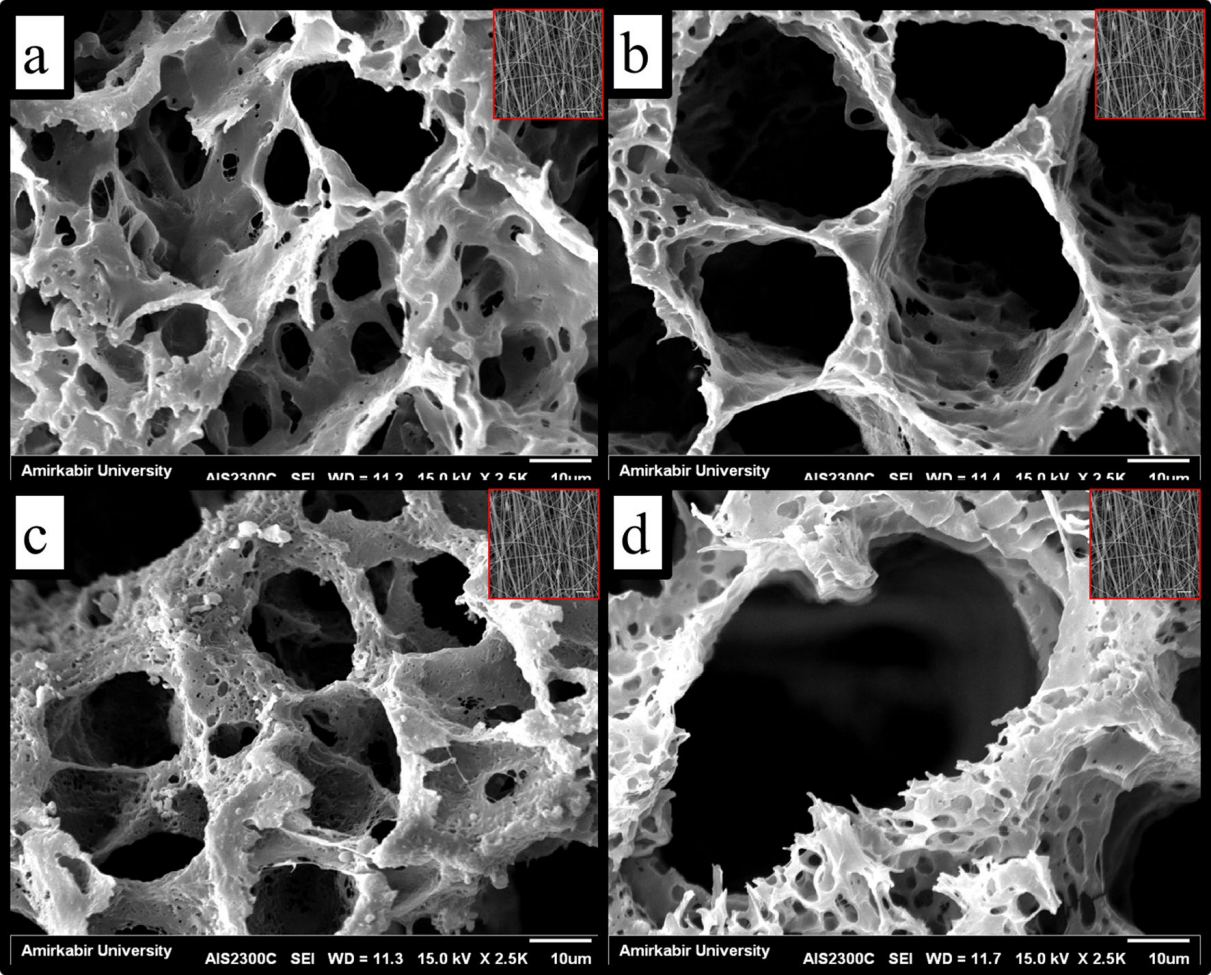
Scanning electron micrographs of the prepared scaffolds. (**a**) PCL/PLLA/GNF, (**b**) PCL/PLLA/GNF/AuNPs (40 ppm), (**c**) PCL/PLLA/GNF/AuNPs (80 ppm), and (**d**) PCL/PLLA/GNF/AuNPs (160 ppm). Insert is SEM image of GNFs.

The synthesized AuNPs have an average diameter of 65.1 nm with a polydispersity index (PDI) of 0.526 measured with DLS. The synthesized AuNPs have zeta potential of + 9.48 ± 0.06 mv, indicating their proper stability. The PDI of 0.526 indicate relatively proper size distribution of the synthesized NPs. SEM image of GNs was shown in Fig. 1 and the nanofibers diameter was 327 ± 183 nm, measured by Image J (1.47v, National Institute of Health, USA) software. Different concentrations of AuNPs (40, 80, and 160 ppm) were added to the scaffolds and their effects on the physicochemical properties and the biological performance were evaluated. The microstructure of the prepared scaffolds was observed using SEM, and the results are presented in Fig. 1. As shown in Fig. 1, the fabricated scaffolds are highly porous (confirmed with porosity measurement assay), which is favorable for cell growth and infiltration. It is also observed that the fabricated scaffolds have interconnected pores, which is vital for the osteogenesis process. The implemented AuNPs can be both adhered onto the surface of matrix and embedded into the polymeric matrix.

The interconnected structure provides cell infiltration and migration, which results in homogenous cell growth and osteogenesis^25^. Moreover, the porous scaffolds with the open and interconnected pores provide vascularization in bone tissue growth, a vital requirement for proper bone tissue regeneration. The prepared scaffolds' pore size was measured using ImageJ software based on the obtained SEM images. The results showed that the pore size of PCL/PLLA/GNF, PCL/PLLA/GNF/AuNPs (40 ppm), PCL/PLLA/GNF/AuNPs (80 ppm), and PCL/PLLA/GNF/AuNPs (160 ppm) were 24.5 ± 177, 44.9 ± 23.6, 53.6 ± 29.7, and 75.8 ± 33.1 µm, respectively. The pore size distribution was uniform in each group. Moreover, the addition of AuNPs (160 ppm) increased the porosity from 81.4 ± 1.69 to 88.1 ± 2.16%, which was not statistically significant (p < 0.05). It is well established that the optimum pore size of a scaffold is in the range of 75–250 μm for bone tissue regeneration^25,26^. Our results showed that the pore sizes of the prepared scaffolds are in the acceptable range. In addition to the pore size, the prepared scaffolds' porosity was measured using the liquid displacement method, and the results are presented in Table 1. The results showed that the scaffolds' porosity is in the range of 80 to 90%, which is acceptable for bone tissue engineering applications. Previous studies showed that the incorporation of NPs increases the porosity of hydrogels. Seyyed Nasrollah et al.^27^ reported that the incorporation of hydroxyapatite crystals increased the porosity of polyurethane scaffold in a dose-dependent manner. They proposed that the incorporation of hydroxyapatite crystals before polymerization influenced the pore generation process.

**Table 1:** The results of the fabricated scaffolds characterization.

	PCL/PLLA/GNF	PCL/PLLA/GNF/AuNPs (40 ppm)	PCL/PLLA/GNF/AuNPs (80 ppm)	PCL/PLLA/GNF/AuNPs (160 ppm)
Porosity (%)	81.4 ± 1.69	83.7 ± 2.03	86.3 ± 3.14	88.1 ± 2.16
Compress modulus (MPa)	8.65 ± 1.18	8.07 ± 0.83	7.51 ± 1.22	7.01 ± 0.15
Contact angle (°)	87.7 ± 1.23	91.2 ± 3.14	95.06 ± 2.17	99.2 ± 2.03
Weight loss (%)				
30 days	30.6 ± 1.77	–	–	27.1 ± 1.44
60 days	41.22 ± 2.13	–	–	35.5 ± 1.87

The porosity of cancellous (or spongy) bone is in the range of 30–90%, and a tissue engineering scaffold must be matched with its porous structure^28,29^. It is known that the proper vascularization and the subsequent osteogenesis take place in the macroporous scaffolds^30,31^. On the other hand, lower porosity promotes cell proliferation and aggregation results in enhanced osteogenesis. Microspores enhance ion exchange for apatite formation and protein adsorption due to the increased surface area, which favors cell growth and bone formation. Macrospores, provide cell infiltration, neovascularization, and proper bone ingrowth^32–35^.

Mechanical properties of the prepared scaffolds were assessed using the compression method according to the ASTM-D5024-95a standard, and the results are presented in Table 1. The results showed that PCL/PLA/GNF without incorporation of AuNPs has the compress modulus of 8.65 ± 1.18 Mpa and the addition of 160 ppm AuNPs decreased the compress modulus to 7. 01 ± 0.15 Mpa. The porosity and pore morphology of biomaterial strangely impact the mechanical properties. The reduced mechanical property with the incorporation of AuNPs can be attributed to the increased porosity. Although the increased porosity compromised the mechanical strength, the obtained compress modulus is in the acceptable range for bone tissue engineering^32,36^.

The hydrophobicity/hydrophilicity of the prepared scaffolds was measured using the water contact angle method. As shown in Table 1, the incorporation of AuNPs increased the water contact angle, indicating the scaffolds' increased hydrophobicity. Lucio et al.^37^ reported that the contact angle of AuNPs is 82.3° ± 8.0° measured using freeze-fracture shadow-casting cryo-scanning electron microscopy. Accordingly, the increased water contact angle from 87.7 ± 1.23 to 99.2 ± 2.03 (°) can be related to the partially hydrophobic nature of the synthesis AuNPs.

Weight loss measurement, as the indication of biodegradation, showed that the fabricated nanocomposites are biodegradable and 41.22 ± 2.13% of mass weight degraded during 60 days. Moreover, it is shown that the addition of AuNPs reduced weight loss from 41.22 ± 2.13 to 35.5 ± 1.87% during 60 days. This observation can be attributed to increased hydrophobicity following the incorporation of AuNPs. Biodegradation is a critical step for a proper scaffold, which makes space for the mature bone tissue formation^38^. The degradation rate of the scaffold must be matched with the healing rate of the injured bone tissue^29^. Kim et al. fabricated magnesium phosphate ceramic 3D scaffolds and observed that, at 6 weeks after implantation, the primary structure of the scaffold was broken, while the scaffold residual remained thick. In another study, Kumar et al.^39^ fabricated load-bearing PCL/poly (lactic-co-glycolic acid) (PLGA)-beta tri-calcium phosphate scaffolds. They observed that the increasing the PLGA concentration accelerated the degradation rate. They reported that, at 6 weeks, all PLGA present in each group was dissolved.

### Cell toxicity and proliferation results

**Figure 2:**
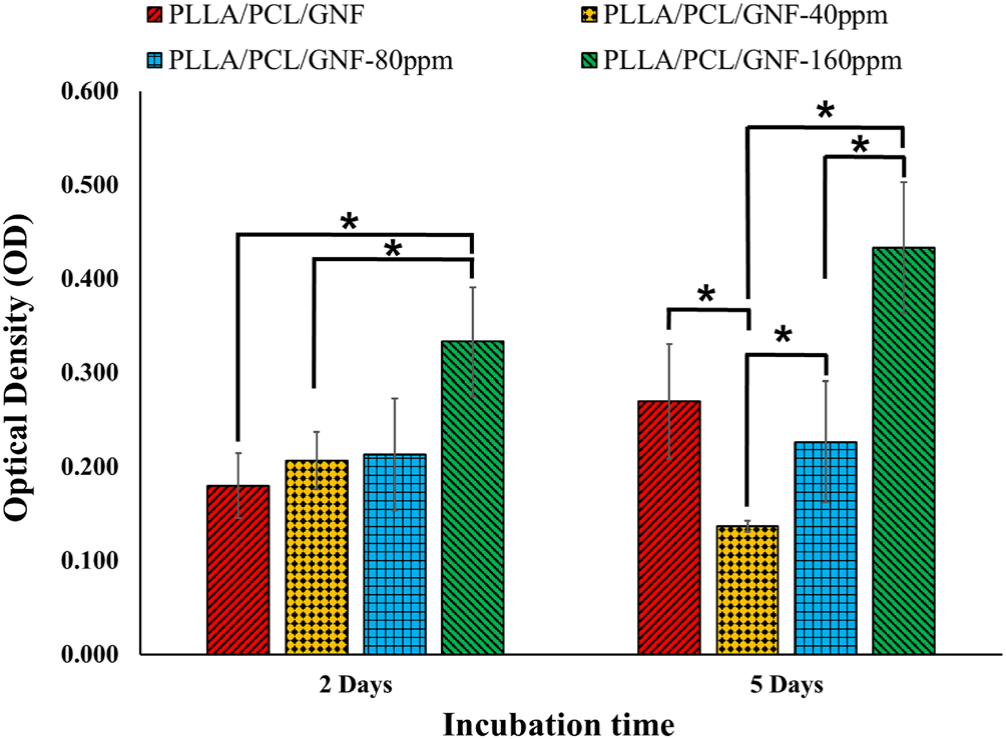
The cytotoxic effects of nanocomposite scaffolds on MG-63 cells measured by lactate dehydrogenase (LDH) assay. MG-63 seeded on the scaffolds with a density of 7000 cells/well in a 96-well plate and incubated for 2 and 5 days. Data represented as mean ± SD, n = 5. *p < 0.05 (obtained by one-way ANOVA).

**Figure 3:**
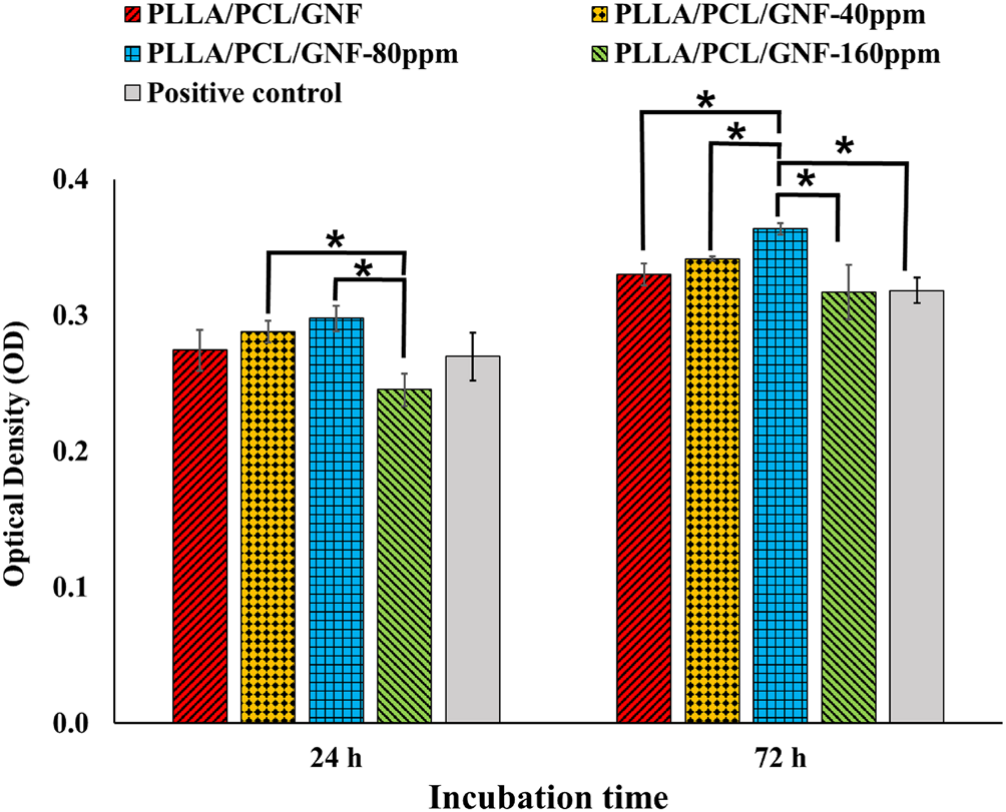
MG-63 cell proliferation on the fabricated scaffolds measured using the MTT assay kit at 24 and 72 h after cell seeding. Control: Tissue Culture Plate (TCP). Data represented as mean ± SD, n = 3. *p < 0.05 (obtained by one-way ANOVA).

Cell toxicity and proliferation were assessed by LDH leakage assay and MTT assay kits, respectively (Figs. 2 and 3). LDH assay is colorimetric method determining the cellular cytotoxicity by measuring LDH enzyme leaked from cell cytosol through the cell membrane.

As shown in Fig. 2, the highest LDH leakage from cells was observed in PLA/PCL/GNF-160 ppm group, whereas the other groups exhibited significantly lower LDH leakage (0.05 < p). Moreover, PLA/PCL/GNF, PLA/PCL/GNF/ AuNPs (40 ppm), and PLA/PCL/GNF/AuNPs (80 ppm) groups exhibited acceptable LDH leakage. These data imply that PLA/PCL/GNF/AuNPs (160 ppm) scaffolds induced cytotoxic effect on cells through the damage to the cell membrane.

The proliferation of MG-63 cells on the fabricated scaffolds was measured using MTT assay (Fig. 3), along with the induced cytotoxicity. The results showed the highest cell growth was obtained by PLA/PCL/GNF/AuNPs (80 ppm) at 72 h, which was statistically significant compared with the other group. Moreover, it was observed that test groups' cell proliferation was higher than the control group, except in PLA/PCL/GNF/AuNPs (160 ppm) at 72 h, which was lower than control. AuNPs are considered as nontoxic and biocompatible structures at the optimum concentrations. Some reports showed the concentration-dependent toxicity of AuNPs. Vecchio et al.^40^ reported concentration-dependent toxicity of AuNPs on *Drosophila melanogaster.* They showed that the observed toxic effects were due to the cellular and molecular damages induced by AuNPs at high concentrations. They proposed that the ROS generated through AuNPs may be responsible for the cellular and genotoxic effects. Carnovale et al.^41^ also reported that AuNPs are biocompatible at the optimum concentration (< 100 µM) and the higher concentration induced toxic effects on cells.

### Animal study results

**Figure 4:**
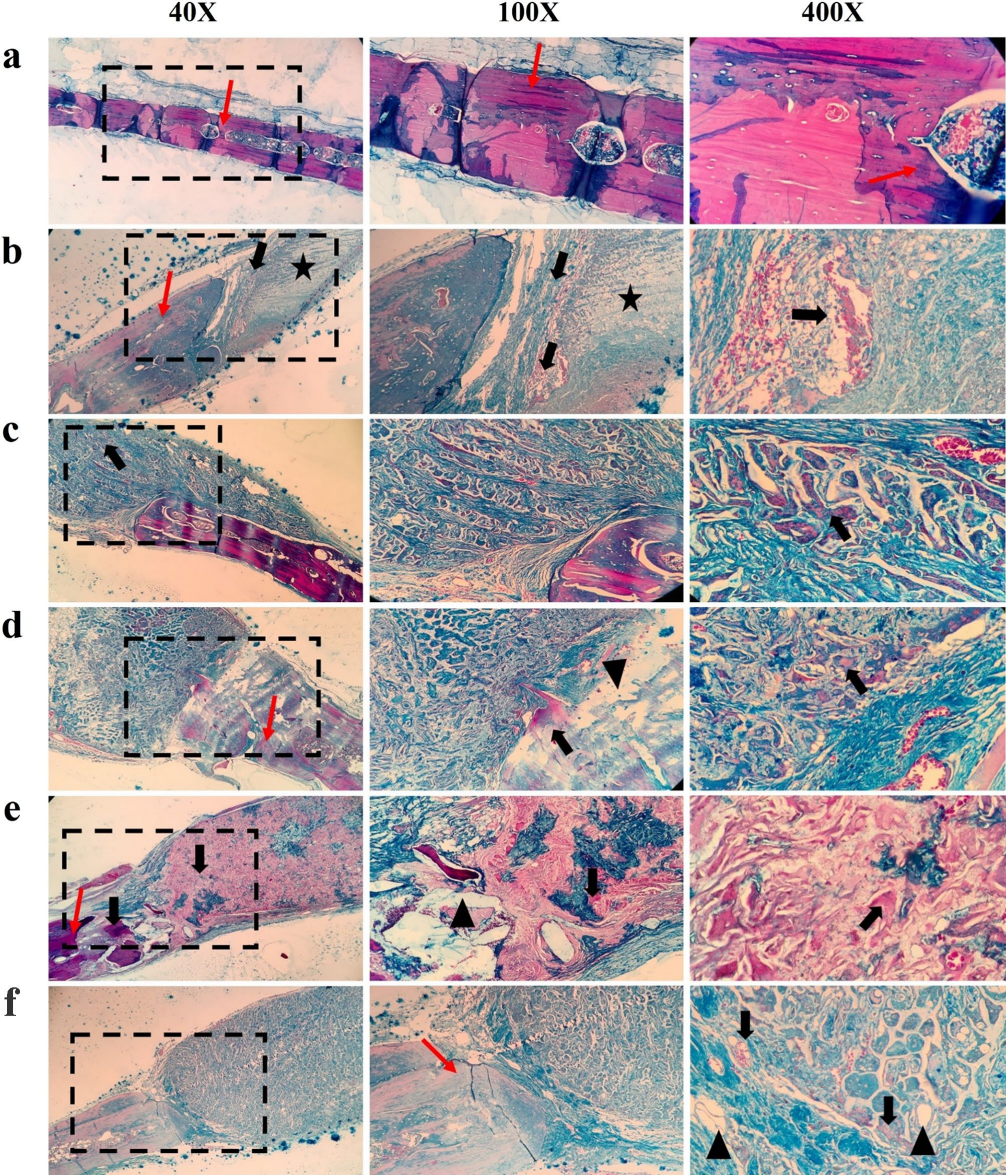
Histopathological sections from the calvaria bone defects and related histomorphometrical analysis (Stained with MTC). (**a**) Positive control, (**b**) Negative control, (**c**) PLLA/PCL/GNF, (**d**) PLLA/PCL/GNF/AuNPs (40 ppm), (**e**) PLLA/PCL/GNF/AuNPs (80 ppm), and (**f**) PLLA/PCL/GNF/AuNPs (160 ppm). *LACT* loose areolar connective tissue (star), *NB* new bone formation (thick arrow), *MB* mature or old bone (red thin arrow), *SR* scaffold remnant (arrowhead).

The bone healing induced by the scaffolds was evaluated in the rat calvarial defect model, and the histopathological and histomorphometric results are presented in Fig. 4 and Table 2, respectively. The results showed that the defect area in the negative control (untreated defect) was filled by a loose areolar connective tissue (LACT) (Fig. 4. star) containing fibroblasts, random immature collagen fibers, and newly-formed blood vessels. The lowest bone ingrowth was also observed in this group. On the other hand, the defect treated with PLA/PCL/GNF/AuNPs (80 ppm) induced higher neo-bone formation (NB) and osteocyte in lacuna (OC) in the defect site. Moreover, the highest degree of NB and woven bone formation, as well as angiogenesis, were observed in this group. Although the scaffold remnants were also visible in the defect area (Fig. 4, arrowhead), they were relatively degraded and almost replaced with new tissues, including collagen fibers, mature bone (MB), and NB. The wettability characteristic of scaffold affects the bone regeneration with different mechanisms. Cells tend to adhere on the hydrophilic substrates and infiltrate into the hydrophilic scaffolds, subsequently regenerate bone tissue^1^. It is reported that the WCA above 90° imply the hydrophobicity and below 90° indicate the hydrophilic nature^42^. Our results showed that, although incorporation of AuNPs increased the WCA value, the resulted data is in the hydrophilic range, below 90°. The in vivo results also showed that the scaffold with the highest WCA value (PLLA/PCL/GNF/AuNPs (160 ppm), WCA of 99.2 ± 2.03°) resulted lower bone regeneration. Accordingly, the lower bone regeneration observed in PLLA/PCL/GNF/AuNPs (160 ppm) could be attributed to the partially hydrophobic nature of the scaffold.

**Table 2:** Histomorphometric findings of bone tissue regeneration in the defect area.

	Negative control	PLA/PCL/GNF	PLA/PCL/GNF/AuNPs (40 ppm)	PLA/PCL/GNF/AuNPs (80 ppm)	PLA/PCL/GNF/AuNPs (160 ppm)
Fibroblast + fibrocyte	62.10 ± 3.55	79 0.42 ± 18.64	93.90 ± 16.03	109.11 ± 22.45	87.09 ± 13.10
Chondroblast + chonrocyte	102.40 ± 5.44	88.50 ± 11.37	53.73 ± 6.74	55.26 ± 4.99	67.32 ± 7.88
Osteoblast + osteocyte	23.25 ± 4.65	51.25 ± 18.09	85.34 ± 16.22	97.60 ± 27.16	76.31 ± 15.05
Osteoclast	3.78 ± 1.66	4.54 ± 1.09	3.31 ± 2.14	2.65 ± 1.28	4.21 ± 0.98
Osteon	3.67 ± 1.27	5.57 ± 1.99	6.93 ± 2.44	7.55 ± 1.69	4.5 ± 1.08

The histomorphometric results showed that the highest fibroblast/fibrocyte, osteoblast/osteocyte, and osteon values were obtained in defect treated with PLA/PCL/GNF/AuNPs (80 ppm). On the other hand, osteoclast cells were observed in this group, indicating more bone regeneration than bone resorption. Bone resorption osteoclast cells is the normal process of debridement after bone injuries, which reabsorb dead bone ends and make space for new bone regeneration^43–45^. These observations revealed that in defect treated with PLA/PCL/GNF/AuNPs (80 ppm), the debridement process has passed and the bone regeneration progressed. The poorest bone regeneration was observed in the negative control group, which exhibited lowest fibroblast/fibrocyte, osteoblast/osteocyte, and osteon values. Among the test groups, the defect treated with PLA/PCL/GNF/AuNPs (80 ppm) showed the highest bone regeneration.

The osteogenic activities of AuNPs reported in several studies^20,22,46–49^. Liu et al. evaluated the osteogenic effects of AuNPs on murine pre-osteoblast cell line MC3T3-E1 and reported increasing of the ALP activity and expressions of Runx2, BMP-2, ALP and OCN gens. Yi et al. reported that AuNPs can elicit osteoinductive activity on mesenchymal stem cells (MSCs) via activating the p38 mitogen-activated protein kinase (MAPK) pathway^22^. Various studies assessed the size effect of AuNPs on the osteoinductive activity. Wan-Kyu et al. reported that 30 nm and 50 nm AuNPs were more potent osteoinductive than smaller (15 nm) or bigger (100 nm) AuNPs^50^. Tsai et al. showed that 10 nm AuNPs did not exhibit osteogenic activities on MG63 osteoblast-like cells^49^. These results proposed that AuNPs in the size range of 30–100 nm are more beneficial for bone tissue engineering applications.

## Conclusion

As an innovative and multidisciplinary approach, tissue engineering has been emerged to eliminate the limitations of conventional tissue regeneration methods. Scaffolds play a central and critical role in tissue engineering approaches and should mimic the native structure of healthy tissue as much as possible. Since bone is a natural nanocomposite, plenty of researches have been conducted to develop nanocomposite-based tissue engineering scaffolds. In the current study, we fabricated a 3D scaffold-based on PCL/PLA polymers through the TIPS method and composited the scaffold with GNFs and AuNPs. The prepared scaffolds were thoroughly characterized in terms of morphology, porosity, hydrophobicity/hydrophilicity, pore size, mechanical properties, and biocompatibility. The results showed that the fabricated nanocomposite possesses properties beneficial for bone tissue engineering. Although biodegradation is a preferred property for bone tissue engineering scaffolds and the inclusion of AuNPs within the scaffold nanocomposite seems at odds with this, the studies have shown that AuNPs do not affect the osteogenesis activities of bone cells^49^. Moreover, there are some reports on the biodegradation of AuNPs within cells^51–53^. It is reported that the observed degradation can be attributed to NADPH oxidase producing a high amount of ROS in the lysosome. Furthermore, we applied low amount of AuNPs (80 ppm) in the scaffolds, which did not disturb the normal functions of cells (confirmed by hemocompatibility, cytocompatibility and animal studies). The animal study showed that PLA/PCL/GNF/AuNPs (80 ppm) scaffold induced the highest bone regeneration. For the future direction, further studies are required to clarify the fate and clearance of the applied AuNPs from within the cells or wound site. This study depicts that the combination of 3D scaffolds with zero (AuNPs) and one (GNFs) dimensional nanostructures can mimic the native structure of bone and promote the bone healing process.

## Materials and methods

### Materials

Gold(III) chloride trihydrate (HAuCl_4_.3H_2_O, 99.9%), gelatin powder (bovine skin, type B), poly (l-lactic) acid (PLLA, Mw = 60 kDa) and poly (ε-caprolactone) [PCL; Mw = 48–90 kDa] were purchased from Sigma-Aldrich (St. Louis, MO). Sodium borohydride (NaBH_4_, 99%), cetyltrimethylammonium bromide (CTAB, 97%), acetic acid, 1,4-Dioxane, and Dimethyl sulfoxide (DMSO) were purchased from Merck Chemicals (Darmstadt, Germany). Fetal Bovine Serum (FBS), Penicillin–Streptomycin (Pen-Strep), MTT ((3-(4, 5-dimethylthiazol-2-yl)-2.5-diphenyl-tetrazolium bromide), Dulbecco's Modified Eagle Medium: Nutrient Mixture F-12 (DMEM/F-12), and Trypsin–EDTA were purchased from Gibco (Germany). Ketamine and Xylazine were obtained from Alfasan (Woerden, Netherlands). MG-63 cell line was obtained from the National Cell Bank of Iran (NCBI), Pasteur Institute of Iran, Tehran, Iran. Male adult Wistar rats were kindly provided by Shahroud University of Medical Sciences, Shahroud, Iran.

### Synthesis of AuNPs

A chemical reduction method was used to synthesis negatively-charged AuNPs based on the previously described studies^54,55^. Briefly, a mixture containing an aqueous solution (12 mL) of HAuCl_4_.3H_2_O (0.5 mM) and 0.5 ml of sodium citrate (10 mM) was stirred for 15 min. Then, 50 µL of fresh and ice-cold sodium borohydride (0.1 M) was added to the prepared solution and mixed well for 2 h. Finally, a 50 kDa centrifugation filter tube was used to wash the synthesized AuNPs and the resulted nanoparticles were centrifuged at 3000 rpm and 4 °C for 4 min to be concentrated.

### Gelatin nanofibers (GNFs) fabrication

Gelatin nanofibers (GNFs) were fabricated based on the electrospinning technique. In this regard, gelatin powder (bovine skin, type B) was dissolved in acetic acid aqueous solution [75% (v/v)] to obtain a 40% (w/v) solution. The electrospinning process was conducted using a commercial electrospinning apparatus (Fanavaran Nano-Meghyas, Tehran, Iran). The fabricated solution was loaded into a 10-mL disposable syringe. The syringe was mounted onto the sample holder. The nozzle, a blunted 20-gauge stainless needle, was connected to the high voltage power supply. The operating parameter, including applied voltage, the flow rate, and nozzle to collector distance, were set as 20 kV, 0.40 mL/h, and 15 cm, respectively. The produced nanofibers were collected from the aluminum wrapped collector and cross-linked using vapor of 20% (v/v) glutaraldehyde at 37 °C for 6 h. The prepared nanofibers were stored in a nitrogen tank overnight, and then crushed into small pieces (GNFs).

### Preparation of PCL/PLLA/GNFs/AuNPs scaffolds

A proper amount of PLLA and PCL powders were dissolved in 1,4-Dioxane and stirred for 12 h to obtain the solution concentration 5% (w/v) of each polymer. The produced polymers solution was mixed with the PCL/PLLA mass ratios of 50/50 and stirred for another 6 h. In the next step, the fabricated GNFs was added to the PCL/PLLA solution and dispersed using vigorous stirring to obtain 10% (w/w) GNFs to PCL/PLLA. Finally, various concentrations of the synthesized AuNPs (40 ppm, 80 ppm, and 160 ppm) were added to the PCL/PLLA/GNFs and further stirred for 24 h. The resulted solutions were transferred and stored at – 80 °C overnight and subsequently freeze-dried at − 54 °C for 48 h using a freeze-drier (Telstar, Terrassa, Spain).

### Characterization of the scaffolds

#### Scanning electron microscopy (SEM) analysis

The microstructure of the prepared PCL/PLLA/GNF and PCL/PLLA/GNF/AuNPs scaffolds were observed by means of a scanning electron microscope (SEM; KYKY Technology Development, Beijing, China) at an accelerating voltage of 20 kV after coating with gold for 250 s using a sputter coater (SCD 004, Balzers, Germany).

#### Contact angle measurement

The hydrophilicity/hydrophobicity nature of the fabricated scaffolds was measure based on the sessile drop technique using a contact angle measuring system (G10, KRUSS, Germany).

#### Weight loss measurement

The prepared scaffolds' degradation rate was measured based on their weight loss in Phosphate Buffer Solution (PBS) (pH 7.4) during 60 days. The samples were finely cut into the shape of the disc (height and diameter of 20 mm and 10 mm, respectively), carefully weighted, totally immersed in tubes filled with 10 mL of PBS, and incubated at 37 °C for 60 days. At the specific time points, specimens were extracted, totally dried, weighed, and the weight loss calculated using Eq. (1).

1 $${\text{Weight~loss}}\,\left( {{\% }} \right) = \frac{{W_{0} - W_{1} }}{{W_{0} }} \times 100$$

where “W_0_” is the initial weight of scaffolds and “W_1_” is the weight of the dried samples after removing them from the media.

#### Mechanical properties

The prepared scaffolds' mechanical properties were measures based on the compression strength method according to the ASTM-D 5024-95a standard with a mechanical testing machine (Santam, Karaj, Iran). In this experiment, dry cylindrical samples of each scaffold (height and diameter of 20 mm and 10 mm, respectively) were analyzed at a cross-head speed of 0.5 mm/min at room temperature and compressed up to 75% original height. Data are described as an average of five test specimens with standard error.

#### Porosity assessment

The porosity of the prepared scaffolds was measured using the liquid displacement technique. Briefly, the initial volume of ethanol was measured (V_1_), 20 mg of each scaffold was immersed into the ethanol for one hour and the volume was measured (V_2_), and finally, the scaffold was removed from the ethanol and the volume was measured (V_3_). Equation (2) was used to calculate the porosity:

2 $${\text{Porosity }}\left( \% \right) = \frac{{V_{1} - V_{3} }}{{V_{2} - V_{3} }} \times 100$$

where V_1_ is the initial volume of 96% ethanol, V_2_ is its volume after soaking of the scaffold in ethanol and V_3_ is the volume of the ethanol after the scaffold removal.

#### Toxicity evaluation

Cell culture studies were performed using MG-63 cell lines and the MTT assay kit was used to quantitatively measure the cell proliferation rate. The prepared scaffolds were cut spherically and put in the bottom of the 96-well plate under sterile conditions. MG-63 cells were cultured at the density of 7 × 10^3^ cells on the scaffolds in DMEM/F12 culture media supplemented with 10% (v/v) FBS, 100 unit/ml penicillin, and 100 µg/mL of streptomycin incubated in a humidified incubator at 37 °C with 5% CO_2_. At each time point (1 and 3 days after cells seeding), the culture medium was removed from the 96-well plate and 0.2 mL of MTT (0.5 mg/1 mL DMEM) was added to each well, and the cells were incubated at 37 °C for 4 h in a dark place. Then, the supernatant was discarded and 150 mL DMSO was added to each well to dissolve the formed formazan crystals. After 10 min, 100 μL from each well was transferred to a new 96-well plate and the optical density was read at 570 nm using Anthos 2020 microplate reader (Biochrom, Berlin, Germany).

### In vivo studies

#### Bone defect creation

The animal studies were performed on thirty healthy adult male Wistar rats (3 months old, weighing 220–250 g) obtained from Pasteur Institute (Tehran, Iran). Animal experiments were carried out according to the Kermanshah University of Medical Sciences guidelines and approved by the university's ethical committee. The rats were randomly divided into five groups (6 rats per group): 1—PCL/PLA/GNF scaffold without AuNPs (S-WNPs), 2—PCL/PLA/GNF/ 40 ppm AuNPs scaffold (S-40 NPs), 3—PCL/PLA/GNF/80 ppm AuNPs scaffold (S-80 NPs), 4—PCL/PLA/GNF/160 ppm AuNPs scaffold (S-160 NPs), and 5—Negative control (defect without scaffold). The animals were then anesthetized by the IP injection of Xylazine (Alfasan, Woerden, Holland; 6–8 mg/kg) and Ketamine (Alfasan, Woerden, Holland; 70–100 mg/kg) mixture. Based on our previous study^56^, the spherical critical-sized defect was created in the calvaria (skull) of the rats and the fabricated scaffolds were implanted in the created defect. Briefly, the periosteum was separated and a bone defect with 7 mm diameter was formed by the trephine (Meisinger), at a speed rate of 1000 rpm (the defect site was irrigated with 0.9% physiological saline to prevent bone necrosis by heat). Subsequently, the scaffolds were embedded into the defect area and the periosteum was repositioned and closed with No. 6.0 nylon suture (SUPA medical devices, Tehran, Iran). The skin was closed with No. 3.0 nylon suture (SUPA medical devices, Tehran, Iran).

### Histological analysis

At the specific time point, the animals were sacrificed under anesthesia, and the harvested tissues (implanted sites) were immediately fixed in the 4% buffered formalin for 48 h. The fixed tissues were stained with Masson’s trichrome (MT) staining. A light microscope (Carl Zeiss, Thornwood, USA) equipped with a digital camera (Olympus, Tokyo, Japan) was used to capture the images and an independent pathologist interpreted the results. The histomorphometric analysis was conducted by counting and analyzing fibroblasts, fibrocytes, chondroblast, chondrocyte, osteoblasts, osteocytes, osteoclast in the defect site using computer software (Image-Pro PlusV.6). The evaluation was conducted on 400X images in six microscopic fields and the averages of different indexes (Mean ± SD) were reported.

### Statistical analysis

The statistical analysis was conducted using the SPSS program, v.23 (IBM, Armonk, NY, USA) and the discriminant evaluation using a one-way ANOVA test with Tukey's multiple comparison test (p < 0.05). All experiments were repeated thrice and samples were evaluated in triplicate. The results were expressed as the mean ± standard errors (SE, n ≥ 3), and P < 0.05 was considered as statistically significant in all evaluations.

### Ethical approval

The study was carried out in compliance with the ARRIVE guidelines (https://arriveguidelines.org).

## Data Availability

The datasets generated during and/or analyzed during the current study are available from the corresponding author on reasonable request.
